# Increasing synchronicity of global extreme fire weather

**DOI:** 10.1126/sciadv.adx8813

**Published:** 2026-02-18

**Authors:** Cong Yin, John T. Abatzoglou, Matthew W. Jones, Alison C. Cullen, Mojtaba Sadegh, Juanle Wang, Yangxiaoyue Liu

**Affiliations:** ^1^ Sierra Nevada Research Institute, University of California, Merced, CA, USA.; ^2^ State Key Laboratory of Resources and Environment Information System, Institute of Geographic Sciences and Natural Resources Research, Chinese Academy of Sciences, Beijing, China.; ^3^ Department of Management of Complex Systems, University of California, Merced, CA, USA.; ^4^ Tyndall Centre for Climate Change Research, School of Environmental Sciences, University of East Anglia, Norwich, UK.; ^5^ Evans School of Public Policy and Governance, University of Washington, Seattle, WA, USA.; ^6^ Department of Civil Engineering, Boise State University, Boise, ID, USA.

## Abstract

Concurrent extreme fire weather creates favorable conditions for widespread large fires, which can complicate the coordination of fire suppression resources and degrade regional air quality. Here, we examine the patterns and trends of intra- and interregional synchronous fire weather (SFW) and explore their links to climate variability and air quality impacts. We find climatologically elevated intraregional SFW in boreal regions, as well as interregional synchronicity among northern temperate and boreal regions. Significant increases in SFW occurred during 1979 to 2024, with more than a twofold increase observed in most regions. We estimate that over half of the observed increase is attributable to anthropogenic climate change. Internal modes of climate variability strongly influence SFW in several regions, including Equatorial Asia, which experiences 43 additional intraregional SFW days during El Niño years. Furthermore, SFW is strongly correlated with regional fire-sourced PM_2.5_ in multiple regions globally. These findings highlight the growing challenges posed by SFW for firefighting coordination and human health.

## Teaser

Increasing synchronicity of global extreme fire weather constrains fire suppression coordination and exacerbates air pollution.

## INTRODUCTION

Extreme fire weather, characterized by exceptionally dry, windy, and often warm conditions, increases fire danger by enhancing vegetation’s susceptibility to ignition and promoting fire spread. The rising trend in extreme fire weather (*1*) and concurrent heat extremes (*2*) underscores the growing threat of not only localized but also synchronous fire weather (SFW), or widespread concurrent extreme fire weather. Combined with the expansion of the wildland-urban interface (*3*) and the accumulation of fuels (*4*) in some regions, SFW can amplify the risk of widespread fire activity compared to localized fire weather (*5*). This heightened risk intensifies the demands on fire suppression efforts, worsens air pollution, adversely impacts human health, and further complicates the coordination of firefighting resources (*6*). The 2019 to 2020 Australian bushfires, for example, brought great challenges to the country’s fire response system and led to widespread air pollution across its most densely populated regions, causing smoke-related premature deaths (*7*). Meanwhile, the seasonality of global fire activity creates opportunities for international fire suppression coordination for countries that do not simultaneously experience seasonal fire risks (*8*, *9*). Given the global impacts of fires on human health, carbon emissions, and biodiversity loss, as well as the increasing challenges in fire management, a comprehensive understanding of SFW is needed to enhance regional resilience to widespread extreme fires (*10*, *11*).

The social and ecological consequences of SFW and associated widespread fire activity result from multiple interconnected processes. First, SFW can reduce the efficacy of fire suppression by overwhelming fire management efforts and limiting the response capacity to address ongoing and new fires (*12*). It not only increases the likelihood of simultaneous widespread fire outbreaks, placing immense pressure on the allocation of regional firefighting resources (*13*), but also creates a high-risk working environment that endangers the safety of personnel. Second, SFW can reduce seasonal windows of opportunity for effective coordination of firefighting resources between regions or countries that do not typically experience overlapping fire seasons, such as Australia and the United States (US). Furthermore, widespread fire activity releases pollutants, including PM_2.5_ and ozone, which can be transported hundreds to thousands of kilometers, thereby affecting public health (*14*).

The concept of synchronicity has been applied to studies of weather and climate extremes, often referred to as concurrent climate extremes or spatially compound events (*15*). Certain concurrent climate extremes such as concurrent heatwaves have significantly increased in both frequency and severity (*16*) as a product of climate change and may facilitate SFW. Teleconnections associated with coupled atmosphere-ocean variability, including the El Niño–Southern Oscillation (ENSO), the Indian Ocean Dipole (IOD), and Tropical Atlantic Variability (TAV), can induce concurrent widespread dry and warm conditions in some regions (*17*–*19*), potentially favoring SFW. Despite its growing relevance, research on SFW remains limited to the western US, Europe, and Australia (*5*, *6*, *9*, *20*, *21*). Furthermore, these interconnected processes can elevate SFW both within specific regions and across multiple regions globally, yet they have not been systematically differentiated in existing studies.

Here, we investigate the patterns and trends of SFW and examine their links to climate variability and air quality. We first analyze the trends, seasonality, and interannual variability of SFW within (intraregional) and across (interregional) 14 regions defined by the Global Fire Emissions Database (GFED) (fig. S1). Next, we explore the relationships between SFW and key modes of climate variability, including ENSO, IOD, and TAV. Last, we quantify the connection between SFW and PM_2.5_ concentrations, as well as population exposure to air pollution associated with SFW.

## RESULTS

### Increasing trends in SFW

**Fig. 1.**
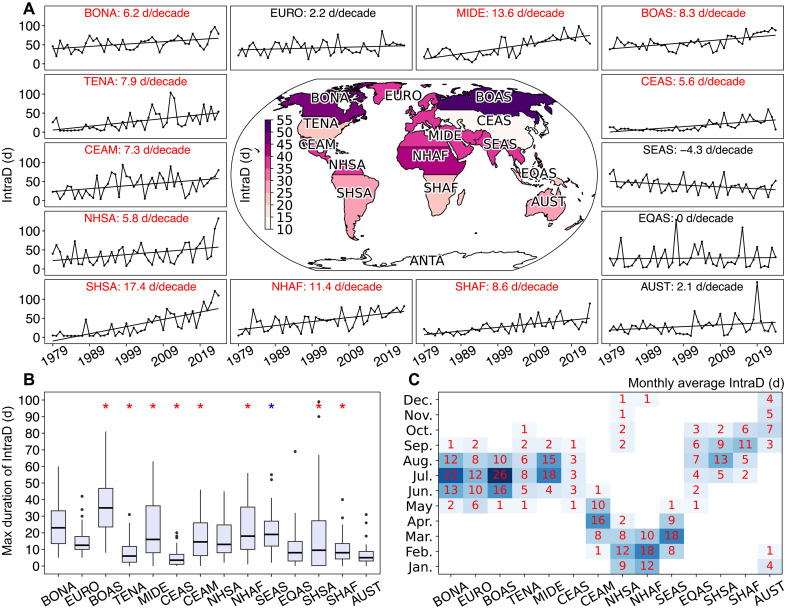
Changes in days with intraregional SFW (IntraD). IntraD is defined as days (d) when FWI exceeds FWI90 across at least 30% of the burnable wildland area within a GFED region on a given day. (**A**) The surrounding plots show the time series of IntraD for each GFED region, with red text indicating significant trends (*P* < 0.05 per modified Mann-Kendall test). The central map depicts the average IntraD from 1979 to 2024. (**B**) Boxplot of the annual maximum duration of IntraD for each GFED region, with red (blue) asterisks (*) indicating significant (*P* < 0.05) positive (negative) trends. (**C**) Monthly average IntraD for each GFED region from 1979 to 2024.

We define days with intraregional SFW when the fire weather index (FWI) exceeds the historical 90th percentile (FWI90) across at least 30% of the burnable wildland area within a GFED region on a given day. The boreal regions experience the highest levels of intraregional SFW due to strong flammability constraints outside the summer months, averaging over 45 synchronous days annually (Fig. 1A). In contrast, Central and East Asia exhibit the lowest levels of synchronicity due to the diverse seasonality in temperature and precipitation patterns within the region. Intraregional SFW significantly increased in 10 of the 14 GFED regions from 1979 to 2024 (*P* < 0.05), with South America exhibiting the largest increase in synchronicity, a trend associated with significant warming and drying (*22*). Conversely, tropical regions such as Southeast Asia exhibit negative trends, likely due to increased atmospheric humidity (*1*). Counterfactual simulations that exclude the first-order influence of climate change on the observational record suggest that anthropogenic climate change (ACC) significantly influences intraregional SFW. Over half of the observed increase in intraregional SFW is attributable to ACC in most regions (fig. S2, A and B). The maximum annual duration of sequential intraregional SFW is generally longer in boreal and tropical regions and has increased across multiple regions from 1979 to 2024 (Fig. 1B). This indicates that extreme fire weather is becoming not only more synchronized but also more persistent, which could further exacerbate fire risk by continuously drying out fuels.

The seasonality of intraregional SFW closely aligns with regional climatological patterns that promote dry fuels. Widespread extreme fire weather in North America, Europe, Boreal Asia, and the Middle East is concentrated between June and August (Fig. 1C), leading to pronounced interregional synchronicity between these regions. This suggests a heightened potential for widespread large fires, increased strain on fire suppression efforts, and greater fire smoke burdens, partially driven by the frequent concurrence of heatwaves and droughts (*23*, *24*). Notably, the US and Australia exhibit limited synchronicity from August to October, facilitating international fire suppression resource sharing with rare seasonal overlaps that occasionally constrain the effectiveness of existing firefighting collaboration networks.

**Fig. 2.**
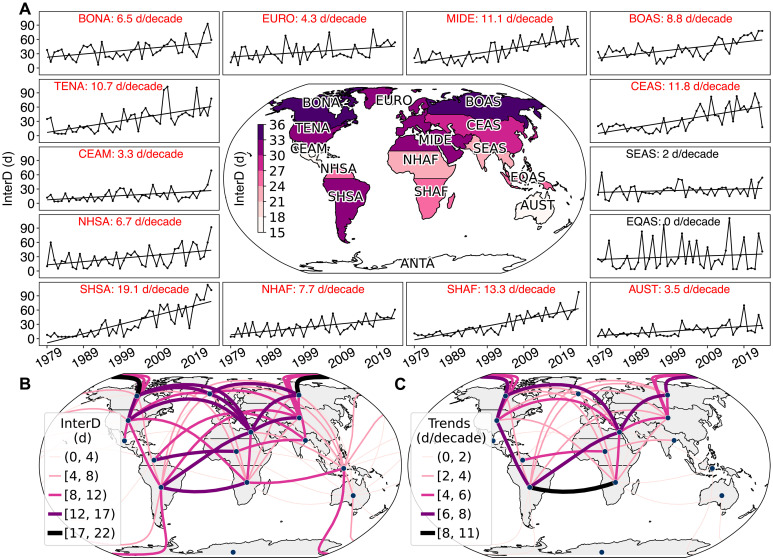
Changes in days with interregional SFW (InterD). InterD is defined as days when the regional average FWI exceeds FWI90 in at least two GFED regions on the same day. (**A**) The surrounding plots show the time series of InterD for each GFED region, with red text indicating significant trends (*P* < 0.05). The central map depicts the average InterD from 1979 to 2024. (**B**) Annual average InterD from 1979 to 2024 between connected GFED regions. (**C**) Significant trends (*P* < 0.05) in InterD between connected GFED regions.

Next, we examine days with interregional SFW, defined as days when the regional average FWI exceeds FWI90 in at least two GFED regions on the same day. North America, Europe, Boreal Asia, the Middle East, and South America experience the highest levels of interregional SFW, with extreme fire weather occurring on the same day in at least one other region for an average of more than 30 days per year (Fig. 2A). In addition, North America and the Middle East, along with South America and Africa, exhibit relatively high interregional SFW, experiencing an average of over 12 synchronous days annually (Fig. 2B). In contrast, extreme fire weather in Australia rarely coincides with that of other regions, reflecting its geographic isolation and distinct fire seasonality, particularly in the temperate parts of the country, such as southern and southeastern Australia, where most of the population resides and fire suppression efforts are concentrated. This separation presents more opportunities for international cooperation in firefighting resource allocation with countries such as the US. Notably, Boreal North America and the contiguous US, regions prone to some of the world’s most catastrophic wildfires (*25*), experience an average of 5 to 15 synchronous days annually (fig. S3A).

Interregional SFW significantly increased in 12 of the 14 GFED regions from 1979 to 2024 (Fig. 2A; *P* < 0.05). In lower- to mid-latitude regions, including South America, Central and East Asia, Africa, and the contiguous US, the annual average number of interregional synchronous days during 2001 to 2024 was three to seven times higher than during 1979 to 2000 (fig. S3B). Interregional synchronous days exhibit a significant positive trend across 45% of GFED region connections (Fig. 2C; *P* < 0.05), with more than half of the increase in nearly all connections attributable to ACC (fig. S2, C and D). The strongest trends are observed between South America and multiple other regions, with the largest increase in SFW across regions occurring between the Southern Hemisphere portions of South America and Africa, which together account for nearly half of global biomass combustion by fires (*26*). The increasing SFW across the US, Australia, Canada, and Europe shortens the window for firefighting coordination, potentially straining existing international firefighting cooperation efforts. For example, a widespread heat dome and chronically dry conditions during the summer of 2021 led to elevated fire weather and a prolonged period during which national fire suppression resources in both the US and Canada were heavily committed, limiting cross-country resource sharing (*27*).

**Fig. 3.**
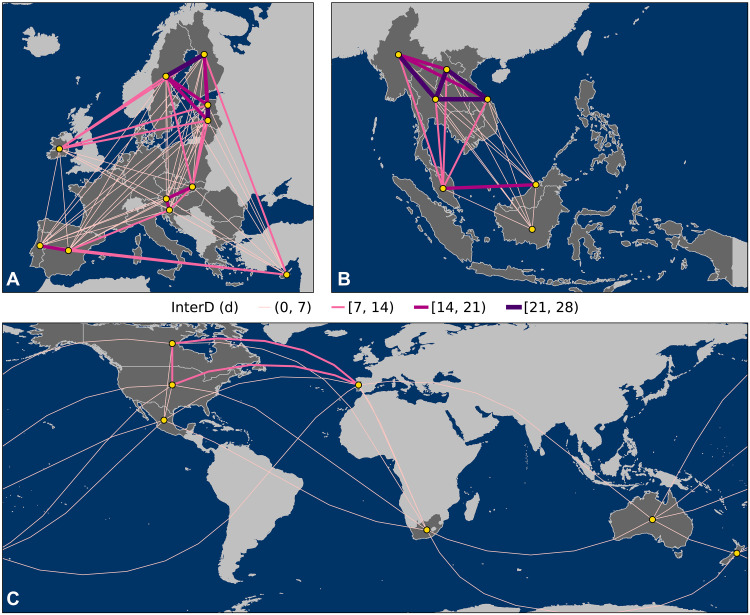
Annual average of intercountry SFW. Annual average InterD from 1979 to 2024 between (**A**) countries in the European Union (EU), (**B**) countries in the Association of Southeast Asian Nations (ASEAN), and (**C**) the US, Canada, Mexico, Australia, New Zealand, Portugal, and South Africa. The unconnected countries represent either zero InterD or insufficient burnable wildland areas per the vegetation layer (fig. S1). Countries outside of the intercountry network for shared fire suppression resource are masked in light gray.

The increase in SFW poses challenges for firefighting cooperation networks across the European Union (EU), the Association of Southeast Asian Nations (ASEAN), and fire-prone countries such as the US, Canada, and Australia (*28*–*30*). For instance, Portugal and Spain experience extreme fire weather on the same day for 19 days per year on average (Fig. 3A), with a 3 days per decade increase in intercountry SFW (fig. S4A). While ASEAN countries have also faced elevated intercountry SFW (Fig. 3B), trends are not significant during 1979 to 2024 (fig. S4B; *P* < 0.05). Moreover, the window for bilateral cooperation among the US, Canada, Mexico, Australia, New Zealand, Portugal, and South Africa may be constrained by the increasing intercountry SFW (Fig. 3C and fig. S4C).

### SFW strongly linked to climate variability and global warming

**Fig. 4.**
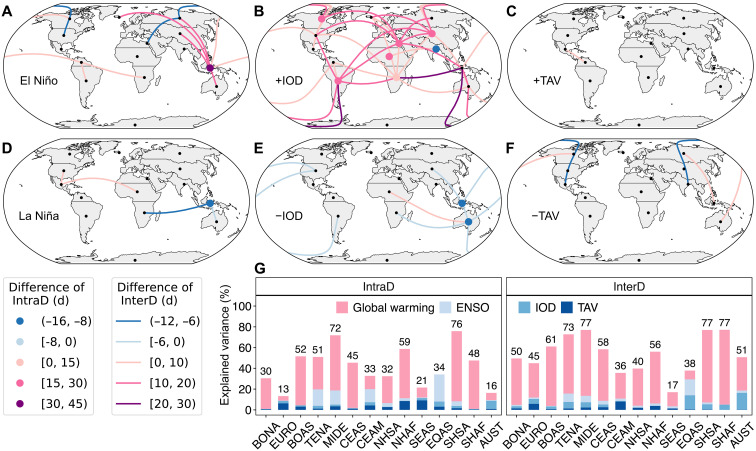
Relationship between SFW, climate variability, and global warming. (**A**) Differences in IntraD (colored points) and InterD (colored lines) between El Niño years and neutral years. Only significant differences (*P* < 0.05 per Mann-Whitney *U* test) are shown. The smaller black points represent the central location of each region. (**B** to **F**) Same as (A), but for positive IOD, positive TAV, La Niña, negative IOD, and negative TAV years, respectively. (**G**) Variance in IntraD and InterD attributed to global warming, ENSO, IOD, and TAV for each GFED region.

Several regions exhibit significant links between SFW and the three coupled atmosphere-ocean modes of variability examined here. For example, during El Niño years, intraregional synchronous days in Equatorial Asia increase by 43 days, while interregional synchronous days between Equatorial Asia and adjacent regions, Australia, Central and East Asia, and Boreal Asia, increase by 10 to 20 days compared to ENSO neutral years (Fig. 4A). Similarly, a widespread increase in interregional synchronous days is observed during positive IOD years (Fig. 4B), whereas the signal is relatively weak during positive TAV years (Fig. 4C). In contrast, during the negative phases of these climate variability modes, intraregional synchronous days decline sharply, accompanied by a decrease in interregional synchronous days (Fig. 4, D to F).

The combined influence of global warming, ENSO, IOD, and TAV explains more interannual variability in interregional SFW than in intraregional SFW during 1979 to 2024 (Fig. 4G). This highlights the role of global warming and large-scale climate variability in driving extreme fire weather synchronicity at broader spatial scales. Among these factors, global warming accounts for the largest share of variability (fig. S5). The warming trend not only intensifies hot and dry conditions locally (*31*) but also increases the likelihood of extreme fire weather occurring simultaneously across regions. These results are generally consistent with our finding that ACC accounts for most of the observed increase in both intra- and interregional SFW (fig. S2). In addition, ENSO has a stronger influence on intraregional SFW, whereas IOD plays a greater role in interregional synchronicity, particularly in regions adjacent to the eastern Indian Ocean, such as Equatorial Asia and Australia. The differential impact of ENSO across regions, along with the moderating effect of the IOD on large-scale climate patterns, helps synchronize extreme fire weather across multiple regions.

### SFW adversely affects air quality

**Fig. 5.**
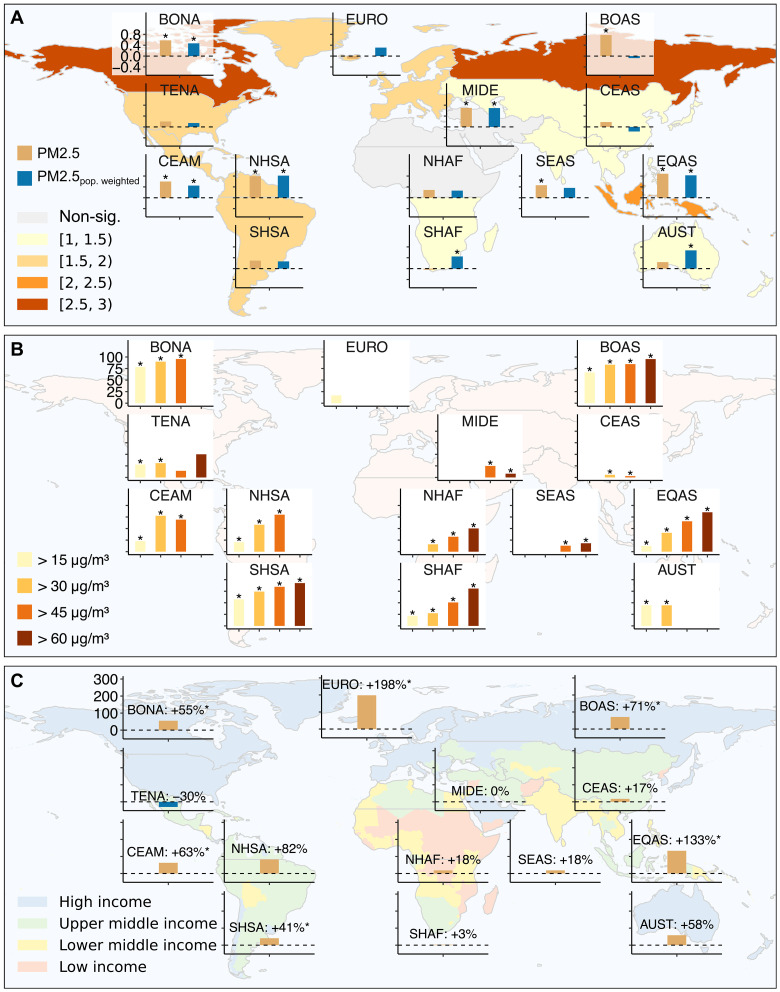
Impact of SFW on air quality. (**A**) Interannual Pearson correlation between IntraD and regional fire-sourced PM_2.5_ concentrations during months accounting for at least 90% of annual IntraD, based on Fig. 1C. Asterisks (*) indicate significant correlations (*P* < 0.05). The colored regions on the basemap depict the ratio of daily average burned area averaged over the GFED region on SFW days to that during the 5 days preceding SFW days. Grey regions indicate non-significant differences between the two groups (*P* ≥ 0.05). (**B**) Proportion of poor air quality days (24-hour all source PM_2.5_ exceeding 15, 45, 50, and 60 μg/m^3^) that coincide with IntraD. Asterisks (*) indicate cases where co-occurrence is not random (*P* < 0.05 per χ^2^ test). (**C**) Additional population exposed to fire-sourced air pollution (annual average all-source PM_2.5_ > 5 μg/m^3^, with fire-sourced PM_2.5_ contributing >50%) during the top 25% years with the highest SFW days, compared to other years. Asterisks (*) indicate significant differences between the two groups (*P* < 0.05).

We examine the impact of SFW on air quality by analyzing the interannual correlation between intraregional SFW days and regional fire-sourced PM_2.5_ concentrations across GFED regions. We find that intraregional SFW significantly degrades air quality in multiple regions (*P* < 0.05) due to elevated burned area during SFW compared with conditions immediately before SFW (Fig. 5A). For example, in Boreal North America, the daily average burned area on SFW days is 2.7 times that of the 5 days preceding SFW. Intraregional synchronous days show a strong correlation with population-weighted PM_2.5_ concentrations in Equatorial Asia, northern South America, and Australia, highlighting the potential for elevated human health risks from fire smoke.

In addition, SFW tends to be more strongly associated with poorer air quality in boreal regions, Equatorial Asia, Africa, and South America. In Boreal Asia, for example, 67% of poor air quality days, defined as days when 24-hour all-source PM_2.5_ exceeds the World Health Organization (WHO) standard of 15 μg/m^3^, coincide with intraregional synchronous days. When this threshold is quadrupled to 60 μg/m^3^, fraction of extremely poor air quality days occurring during SFW rises to 95% (Fig. 5B), underscoring the role of SFW in driving large-scale fires that contribute to severe air pollution.

Furthermore, intraregional SFW is significantly linked to population exposure to fire-sourced air pollution, defined as populations residing in areas where annual average all-source PM_2.5_ concentrations exceed the WHO standard of 5 μg/m^3^, with fire-sourced PM_2.5_ contributing more than 50%. In Europe, during the top 25% years with the highest SFW days, population exposure is 198% higher than in other years (Fig. 5C). Regions experiencing greater additional population exposure tend to have middle to higher income levels, which may enhance their resilience to the severe health impacts of widespread large fires driven by SFW.

## DISCUSSION

By investigating SFW within and across global regions, we identify boreal regions as hotspots for widespread concurrent extreme fire weather. These patterns are strongly linked to regional climatology (*32*). Specifically, boreal regions are predominantly influenced by an extensive Subarctic climate, which aligns temperature and precipitation patterns across multiple regions, constraining flammability during the cool season and increasing the synchronicity of relatively warm and dry conditions that contribute to elevated fire weather. By contrast, regions with limited SFW, such as Central and East Asia, span humid subtropical, temperate, continental, and arid climates, which lack seasonal coherence in the periods favorable for fuel drying. Extreme fire weather is highly synchronized between North America, Europe, Boreal Asia, and the Middle East, as well as between South America and Africa, whereas Australia remains relatively distinct from other regions. This synchronicity is primarily driven by the concurrence of warm and dry conditions. Our findings partially reflect prior work that shows tropical regions and the Northern Hemisphere exhibiting elevated concurrent heat extremes, while Australia rarely experiences synchronicity with other regions (*2*, *16*, *23*).

We find a significant positive trend in SFW within and across most regions, along with a prolonged duration in multiple regions. Counterfactual simulations suggest that the majority of the observed increase in intra- and interregional SFW is attributable to ACC, complementing other studies that have quantified the role of climate change on rising regional fire risk (*33*, *34*) and global fire impacts (*35*). The increased SFW is consistent with observations in the western US, Europe, and Australia (*5*, *6*, *9*, *20*, *21*) and aligns with the rising concurrence of warm and dry conditions globally (*36*). South America exhibits the most pronounced increase in extreme fire weather (fig. S6), as well as intraregional and interregional SFW. We also observe a strong increase in SFW between South America and Southern Africa, both recognized as major global carbon emission hotspots (*26*). While fire activity in the African savanna has declined significantly in recent years as a product of land use (*37*), the growing synchronicity between fire-prone regions may increase the propensity for years with climate-driven high fire emissions, adding variability that is unlikely to be captured in policy-relevant global carbon budgets due to the poor representation of fire emissions in global models (*38*). In addition, given the higher vulnerability and health burden of populations in these regions, increased SFW poses serious risks to human health through degraded air quality.

Increased SFW highlights the challenges of international firefighting cooperation, including the exchange of fire management expertise, personnel, and equipment. Currently, the European Union, the Association of Southeast Asian Nations, and fire-prone countries such as the US, Canada, and Australia have established bilateral and multilateral firefighting cooperation mechanisms, which have proven effective in managing recent major wildfires (*8*, *9*). However, given that some of these regions already experience overlapping fire seasons, this growing synchronicity may restrict opportunities for international firefighting collaboration, emphasizing the urgent need for more robust and adaptive strategies in global fire management.

Although global warming is the primary driver of interannual variability in SFW during 1979 to 2024, several regions exhibit significant relationships between SFW and three major modes of climate variability. ENSO has a stronger influence on intraregional SFW, whereas IOD plays a greater role in interregional synchronicity. For example, El Niño typically reduces precipitation and raises temperatures in the northern Amazon, intensifying drought conditions and heightening fire risks (*39*). In Equatorial Asia, El Niño years correspond to a significant rise in intraregional SFW, driven by elevated temperatures and pronounced rainfall deficits. The eastward shift of the rain belt during El Niño exacerbates rainfall shortages in western Pacific regions, including Equatorial Asia and Australia, fostering fire-prone conditions (*40*). Furthermore, we observe a widespread increase in interregional SFW between Equatorial Asia and other regions during positive IOD years. Notably, intraregional SFW reached record levels during the 2019 to 2020 Australian fire season, likely driven by strong positive IOD events (fig. S7). Positive IOD events cause sea surface cooling in the eastern Indian Ocean, particularly near Indonesia and northern Australia, which suppresses convection, reduces rainfall, and increases fire potential across large areas of Australia (*41*, *42*).

Regional fire-sourced PM_2.5_ exhibits a stronger correlation with intraregional SFW in Equatorial Asia, Central America, and boreal regions compared to other regions. This is potentially facilitated by more direct links between FWI and burned area in forested lands globally (*43*) and higher unit emissions from woodland and forest fires than savanna and grassland fires (*26*). Similarly, we observe a stronger association between intraregional SFW and air pollution, as well as related population exposure, in boreal and tropical regions. These findings underscore the significant health risks posed by SFW, which can trigger widespread fires and elevate population exposure to air pollution.

The limitations and uncertainties of this study remain in the following aspects. First, the results may partially be impacted by the selection of spatial zonings (i.e., the use of GFED regions). For example, patterns and trends in certain regions with high SFW, such as the western US, may be obscured due to the large regions used in this study. We note that the herein proposed approach can be used at a variety of spatial scales, including at the country-level (e.g., Fig. 3 and fig. S4). Second, fire activity is influenced by a combination of weather, fuel, ignition, and suppression factors (*43*, *44*). Consequently, SFW does not necessarily translate into widespread large fires and associated air pollution. Future research should focus on finer spatial and temporal scales to better capture localized fire dynamics and provide actionable information for fire response agencies and the public. For example, seasonal and higher-resolution analyses are essential for accurately identifying SFW across regions with similar fire seasons. Incorporating additional factors, such as vegetation type, land use changes, and human activities, can further enhance the accuracy of fire risk assessments.

These findings provide a scientific foundation for understanding concurrent extreme fire weather and preparing for an increasingly fire-prone future. They also emphasize the compounding effects of SFW on air quality, public health, and fire management. Addressing these challenges requires coordinated international efforts, including the implementation of early warning systems (*45*), enhanced wildfire management strategies, and targeted interventions to reduce population exposure to fire smoke in vulnerable regions.

## MATERIALS AND METHODS

### Data

To identify SFW, we calculate daily FWI at 0.25° resolution for the period 1979 to 2024 using fifth-generation European Centre for Medium-Range Weather Forecasts (ECMWF) Reanalysis data (*46*), based on daily maximum air temperature, daily minimum relative humidity, daily mean wind speed, and daily total precipitation, following prior studies (*47*). We also apply an overwintering procedure in our calculations that accounts for interseasonal drought in cold climates (*48*). To assess the contribution of ACC, we apply the same methodology to derive counterfactual FWI that removes the first-order influence of modeled climate change in the variables used to calculate FWI. The counterfactual is constructed by subtracting the low-pass filtered signal of monthly changes in temperature, humidity, wind speed, and precipitation relative to a quasi-preindustrial climate (1850 to 1900) as simulated by the multimodel mean of 20 models participating in the Coupled Model Intercomparison Project Phase 6 (table S1) from the observational record, following prior studies (*49*, *50*). The FWI is the most widely used fire danger index globally due to its effectiveness across various biomes (*44*), integrating fuel dryness and potential fire spread driven by meteorological conditions. To extract FWI data for burnable wildland areas (forests, mixed cover, woodlands, shrublands, and grasslands), we exclude croplands, barren land, and urban or built-up areas using a constant Global Land Data Assimilation System (GLDAS) vegetation map at 0.25° resolution (*51*), matching the scale of the FWI data. Vegetation dynamics during the study period are not considered.

We adopt the GFED zoning approach (*52*) to investigate SFW within (intraregional) and across (interregional) regions, which is widely used in large-scale fire studies (*44*, *53*, *54*). This subcontinental zoning approach divides the world’s land area into 14 regions (fig. S1), considering fire patterns, broad biomes, and administrative boundaries. It not only adds importance to the investigation of fire response to SFW across biomes but also provides a reference for fire management across administrative entities. We choose GFED regions as our primary zoning approach given their widespread usage in prior studies and the tractable number of regions for analyses. However, we note that the approaches applied here can be applied to other geographic units.

To investigate the links between SFW and major modes of climate variability (*55*), we identify the positive and negative phases of ENSO, IOD, and TAV (fig. S8) using standardized anomalies (±0.8σ) of detrended sea surface temperature (SST) (*56*) relative to the 1991 to 2020 climatological mean. SSTs are first detrended by subtracting a 20-year moving average to isolate interannual variability. For ENSO, we use monthly SST anomalies averaged over the Niño 3.4 region (170°W–120°W, 5°S-5°N). The IOD phase is defined on the basis of July-September SST anomalies using the dipole mode index, calculated as the difference between the western Indian Ocean (50°E-70°E, 10°S-10°N) and the eastern Indian Ocean (90°E-110°E, 10°S-0°N). For TAV, we analyze April-July SST anomalies over the tropical Atlantic basin (60°W-20°E, 20°S-20°N).

We retrieve PM_2.5_ concentration and burned area data to investigate the relationship between SFW and air pollution. Daily gridded all-source PM_2.5_ concentrations at 0.75° resolution for the period 2003 to 2024 are obtained from the ECMWF Atmospheric Composition Reanalysis 4 (*57*). Monthly gridded fire-sourced PM_2.5_ concentrations at 0.25° resolution for the period 2000 to 2019 are also acquired (*58*), derived using machine learning and chemical transport models, and validated against station observations to ensure consistency and reliability. We calculate daily gridded burned area at 0.25° resolution based on the MCD64A1 product (*59*), which provides burn date information at 500 m resolution. Burned grid points are identified and burned area is aggregated at 0.25° resolution for each day during 2002 to 2023. We then calculate the ratio of daily average burned area on intraregional SFW days to that during the 5 days preceding SFW to assess whether elevated PM_2.5_ concentrations are associated with increased burned area during SFW.

We use global gridded population data at 0.25° resolution for the years 2000, 2005, 2010, 2015, and 2020 (*60*) to estimate human exposure to air pollution associated with SFW. These population data are generated using a proportional allocation gridding algorithm incorporating approximately 13.5 million national and subnational administrative units. The results align closely with national censuses and population registers, ensuring accuracy and representativeness.

### Intraregional and interregional SFW

SFW refers to the occurrence of spatially compounding extreme fire weather. Building on studies focused on the western US (*5*), which define SFW as FWI exceeding the 90th percentile (FWI90) across at least 40% of forested land, we define intraregional SFW days (IntraD) as those when the FWI exceeds FWI90, based on the 1991 to 2020 reference period, across at least 30% of the burnable wildland area within a GFED region. The adjustment from 40 to 30% accounts for the larger regions examined in this study, where a higher threshold may hinder the detection of extreme fire weather.

Similarly, drawing on the concept of concurrent heat extremes (*16*), we define interregional SFW days (InterD) as those when the regional average FWI exceeds FWI90 in at least two GFED regions on the same day, expanding our understanding of fire weather synchronicity at the global scale.

We also present results based on alternative thresholds to test sensitivity (table S2 and fig. S9). For IntraD: (i) a fixed FWI90 with 20 and 40% thresholds of regional extent and (ii) a fixed 30% of regional extent threshold with FWI95 and FWI99; for InterD: FWI95 and FWI99. The results show that although higher thresholds detect fewer SFW, the spatial patterns and trends remain consistent across thresholds.

### Attributing SFW to global warming, ENSO, IOD, and TAV

We apply the variance decomposition method (*61*) to quantify the contributions of global warming, ENSO, IOD, and TAV to SFW. This method assesses the relative contribution of each independent variable to the overall explained variance ($R^{2}$) in a linear regression model. Specifically, it quantifies the percentage reduction in $R^{2}$ when each variable is removed from the model, isolating its unique contribution. The function begins by fitting a full regression model with all predictors to compute the total $R^{2}$ ($R_{\text{full}}^{2}$). For each predictor, it creates a reduced model by excluding the predictor, calculates the reduced $R^{2}$ ($R_{\text{reduced}}^{2}$), and determines the contribution of the excluded variable as $∆R^{2}=R_{\text{full}}^{2}-R_{\text{reduced}}^{2}$. The contributions are expressed as percentages, calculated as

$${\text{Contribution}}_{i}=\frac{∆R_{i}^{2}}{R_{\text{full}}^{2}}\times 100$$ (1)

where i represents each predictor. This approach provides a clear and interpretable framework for understanding the relative importance of predictors in explaining the variance of the dependent variable.

For this study, we conduct variance decomposition analysis for the period 1979 to 2024. The independent variables include annual indices for global warming, ENSO, IOD, and TAV, represented by global average temperature anomaly (*62*) and standardized SST anomalies, which were also used to identify the phases of ENSO, IOD, and TAV as described in the “Data” section. We remove linear trends in the ENSO, IOD, and TAV indices to isolate the long-term trend associated with global warming. The dependent variable is the annual number of IntraD or InterD for each GFED region.

### Population-weighted PM_2.5_ concentrations

Fire-sourced PM_2.5_ concentrations are weighted by population to estimate human exposure to fire-sourced emissions, ensuring that areas with higher population densities contribute more to the overall PM_2.5_ concentration. The population-weighted PM_2.5_ concentration at each grid point is calculated as the product of the PM_2.5_ concentration at that grid point and the proportion of the population at that grid point relative to the global population. To match the temporal resolution of fire emission data, the annual population data are interpolated to monthly resolution using the nearest-neighbor method.

### Population exposure to fire-sourced air pollution

We assessed population exposure to fire-sourced air pollution to examine the relationship between SFW and human health. Following the methodology outlined by Xu *et al.* (*58*) and in accordance with the 2021 WHO air quality guidelines for long-term exposure risks (*63*), we define fire-sourced air pollution as occurring in areas where the annual average all-source PM_2.5_ concentration exceeds 5 μg/m^3^, with fire-sourced PM_2.5_ contributing more than 50% of the total. Population exposure is then defined as the number of people residing in these areas.

To evaluate socioeconomic disparities in exposure, we use the World Bank’s income-level classifications, categorizing economies into four groups based on gross national income per capita in 2023: low-income economies (≤$1145), lower middle-income economies ($1146 to $4515), upper middle-income economies ($4516 to $14,005), and high-income economies (>$14,005).

### Trend analysis and significance testing

To assess trends in SFW and extreme fire weather (Figs. 1, A and B, and 2, A and C; and figs. S2, B and D, S4, S6, and S9B), we applied the modified Mann-Kendall test to account for serial autocorrelation (*64*). To control the false discovery rate, we applied the Benjamini-Hochberg procedure.

To test the significance of differences between two groups (Figs. 4, A to F, and 5C; and figs. S2, A and C, and S3B), we used the Mann-Whitney *U* test, given the small sample sizes and the likely non-normal, potentially skewed distributions of variables such as IntraD and InterD during 1979 to 2024.

To evaluate whether the co-occurrence of poor air quality days and IntraD exceeded what would be expected by chance (Fig. 5B), we constructed 2 by 2 contingency tables for each GFED region using daily data from 2003 to 2024. Statistical significance of co-occurrence was assessed using the χ^2^ test of independence. All analyses were conducted using Python 3.13.5 and R 4.5.1.
