# Supplementary material for: Increasing synchronicity of global extreme fire weather
Source: Sci Adv. 2026 Feb 18;12(8):eadx8813. doi: 10.1126/sciadv.adx8813 (PMC12915598; doi:10.1126/sciadv.adx8813)
Supplement: Supplementary file 1 — Figs. S1 to S9 Tables S1 and S2 [file sciadv.adx8813_sm.pdf]

Science Advances
AAAS

Supplementary Materials for

Increasing synchronicity of global extreme fire weather

Cong Yin *et al.*

Corresponding author: Cong Yin, congyin@ucmerced.edu

Sci. Adv. **12**, eadx8813 (2026)
DOI: 10.1126/sciadv.adx8813

This PDF file includes:

Figs. S1 to S9
Tables S1 and S2

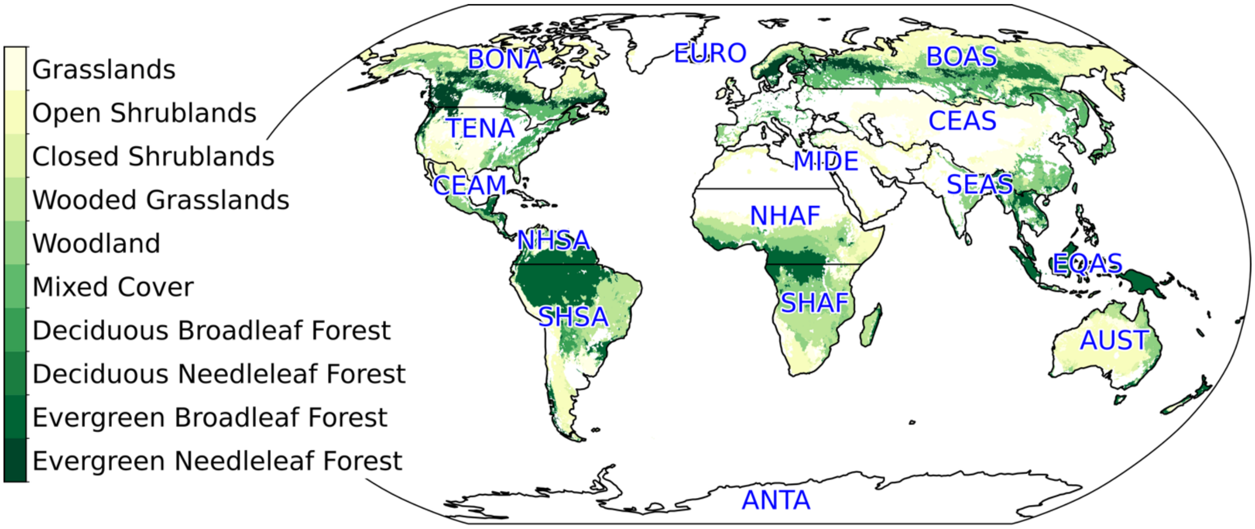

**BONA**	Boreal North America	**SHAF**	Southern Hemisphere Africa	**CEAS**	Central Asia
**TENA**	Temperate North America	**CEAM**	Central America	**SEAS**	Southeast Asia
**NHSA**	Northern Hemisphere South America	**EURO**	Europe	**EQAS**	Equatorial Asia
**SHSA**	Southern Hemisphere South America	**MIDE**	Middle East	**AUST**	Australia
**NHAF**	Northern Hemisphere Africa	**BOAS**	Boreal Asia	**ANTA**	Antarctica

Fig. S1. Study area and vegetation types. Regions as defined by the Global Fire Emissions Database *(52)* and vegetation types as defined by the Global Land Data Assimilation System *(51)*. Unburnable wildland areas, including cropland, barren land, and urban and built-up areas, are excluded and shown in white.

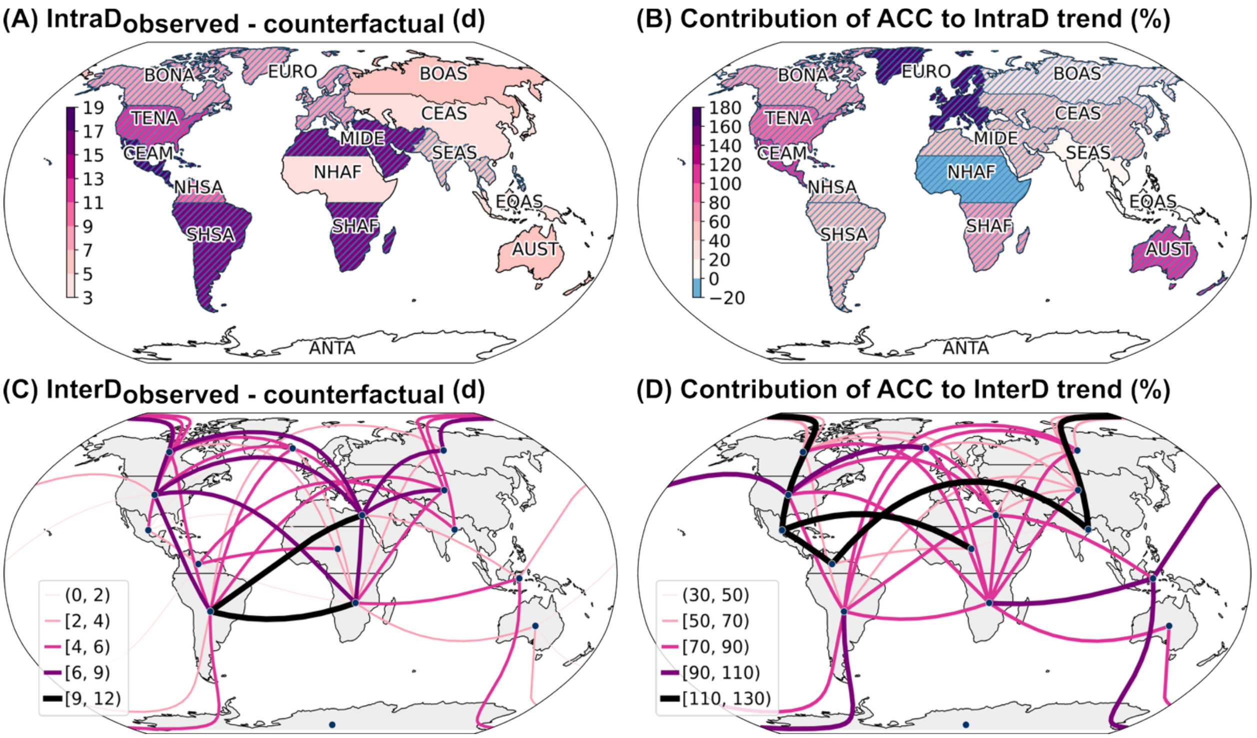

Fig. S2. Contribution of anthropogenic climate change (ACC) to SFW. (A) Difference in average IntraD during 1979–2024 based on observed FWI and counterfactual FWI. Hatched regions indicate significant differences ($p < 0.05$) between the two groups. (B) Trend in IntraD attributed to ACC, quantified as the trend of the difference between IntraD based on observed FWI and counterfactual FWI, expressed as a percentage of the trend in IntraD based on observed FWI. (C) and (D) Same as (A) and (B), but for InterD.

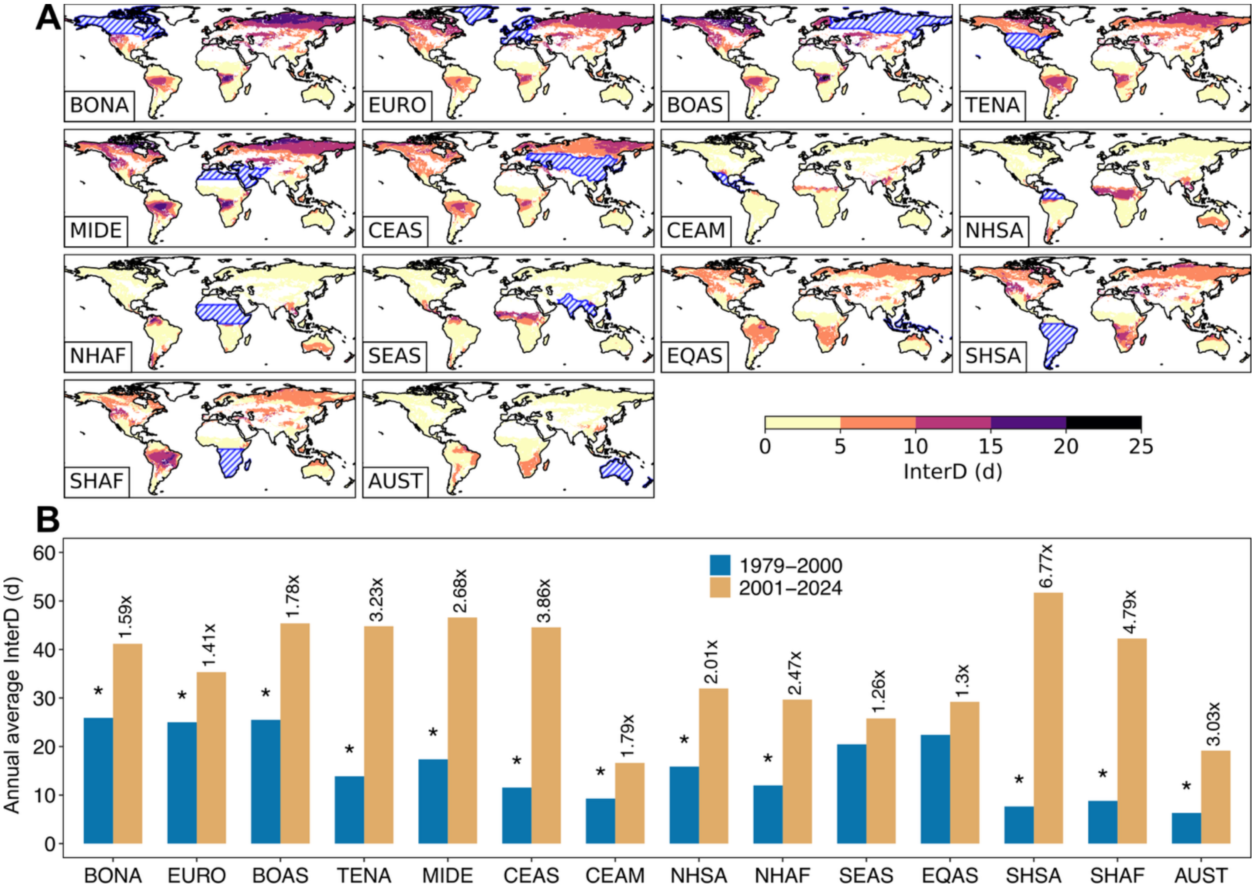

Fig. S3. Changes in InterD. (A) Yearly average InterD from 1979 to 2024 between the highlighted GFED region and the remaining grid points. (B) Yearly average InterD for each GFED region, compared between the periods 1979–2000 and 2001–2024. Asterisks (*) indicate significant differences ($p < 0.05$) between the two time periods.

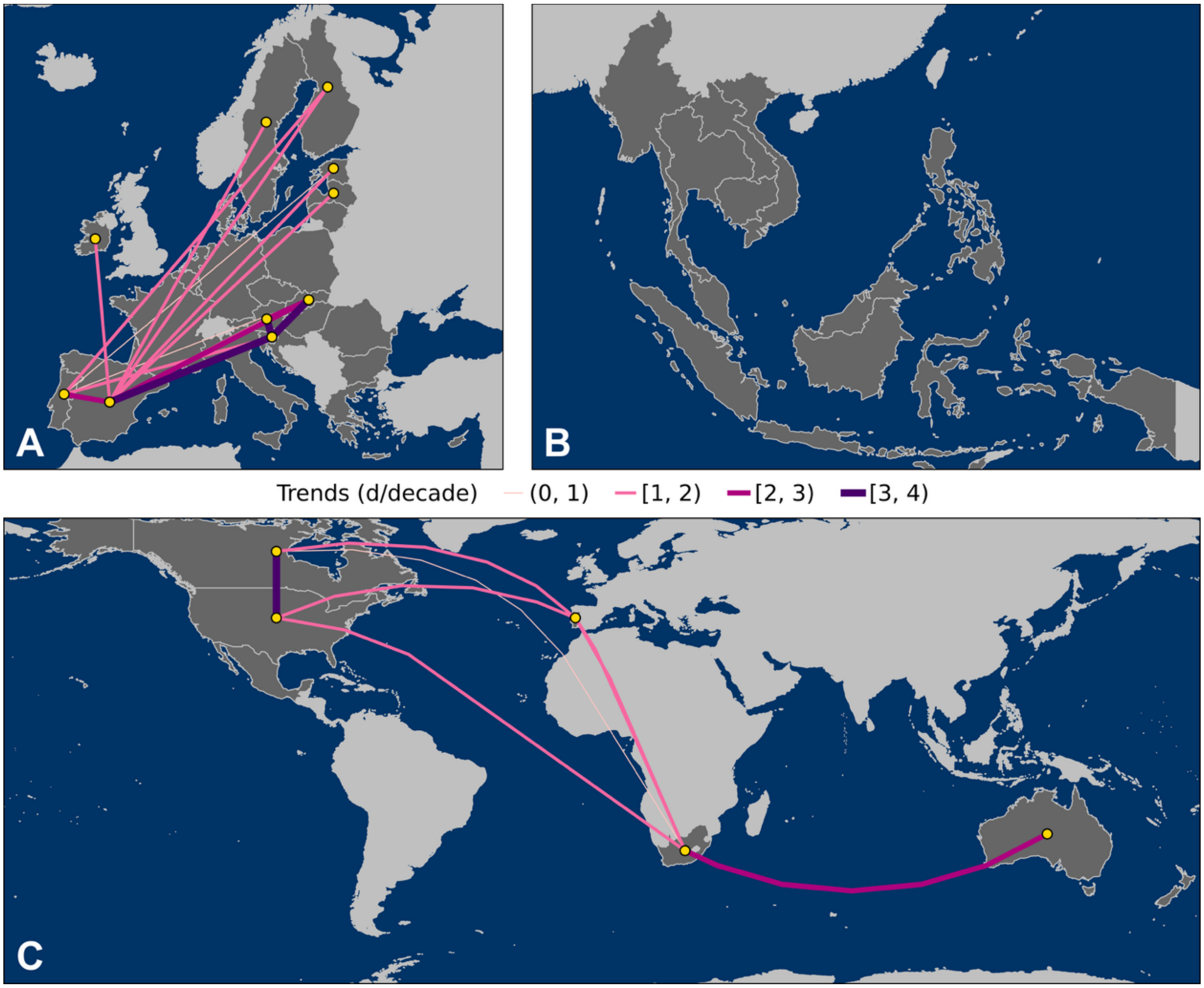

Fig. S4. Changes in inter-country SFW. Significant trends ($p < 0.05$) in InterD between (A) connected countries in the European Union (EU), (B) connected countries in the Association of Southeast Asian Nations (ASEAN), and (C) the United States, Canada, Mexico, Australia, New Zealand, Portugal, and South Africa.

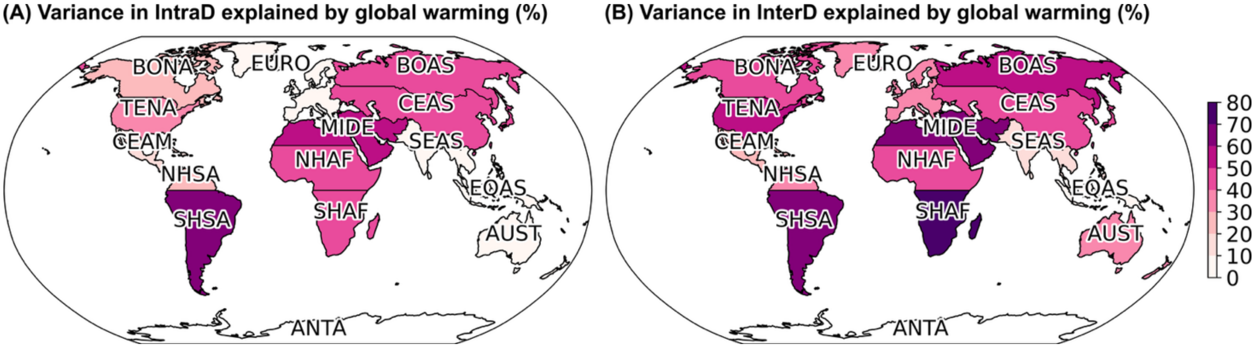

Fig. S5. Global warming accounts for the largest share of variability in SFW. (A) Percentage of variance in IntraD explained by global warming. (B) Same as (A), but for InterD.

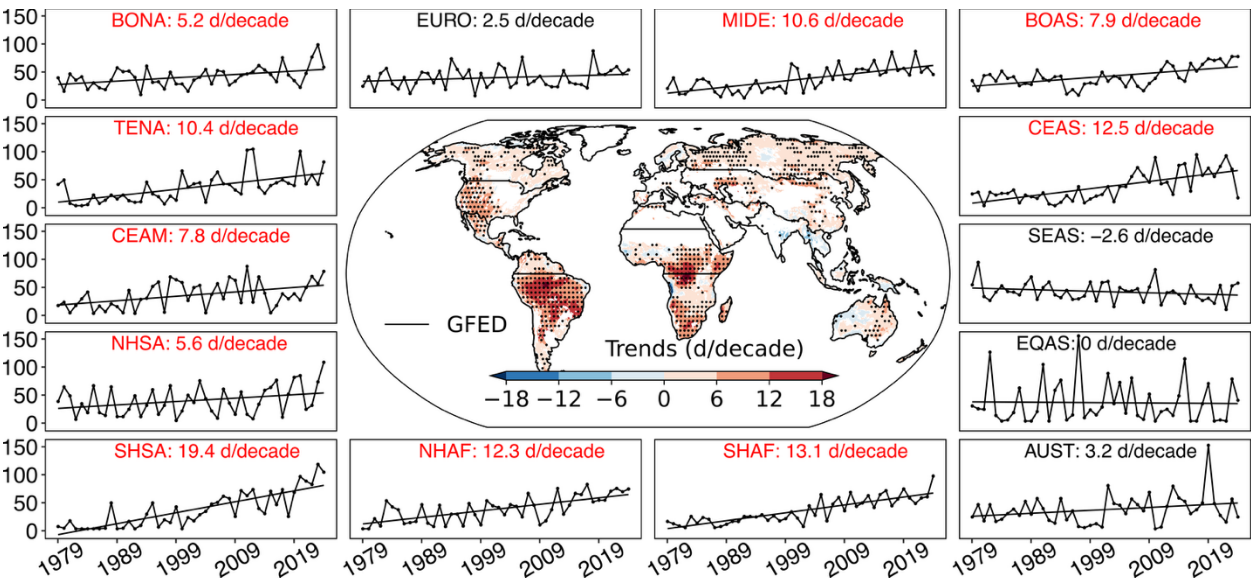

Fig. S6. Changes in days with extreme fire weather. The surrounding plots show the time series of the number of days exceeding FWI90 for each GFED region, with red text indicating significant trends ($p < 0.05$). The central map illustrates the trend from 1979 to 2024, with dots denoting significant trends ($p < 0.05$).

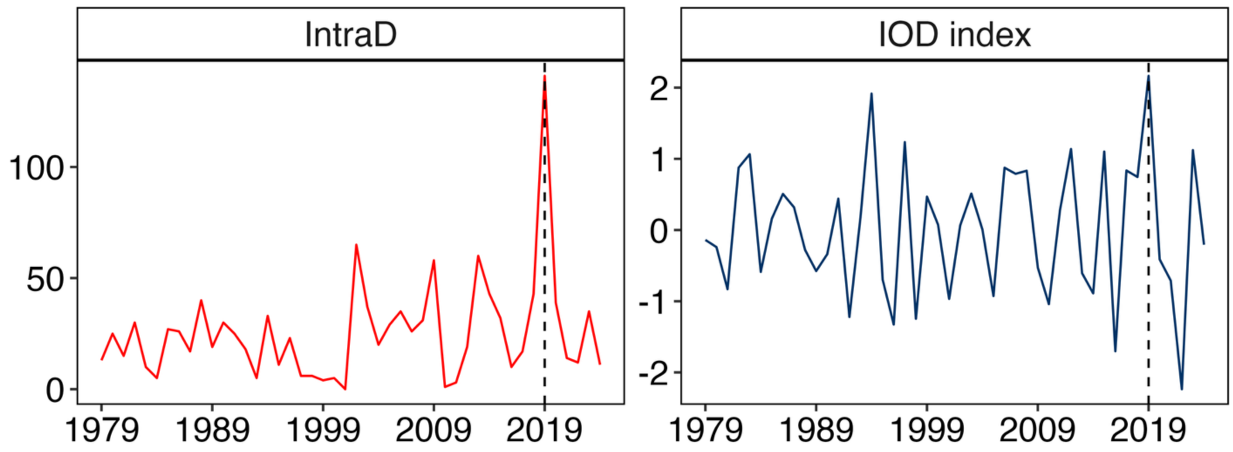

Fig. S7. IOD-driven intra-regional SFW in Australia. Time series of Australian IntraD and the IOD index from 1979 to 2024.

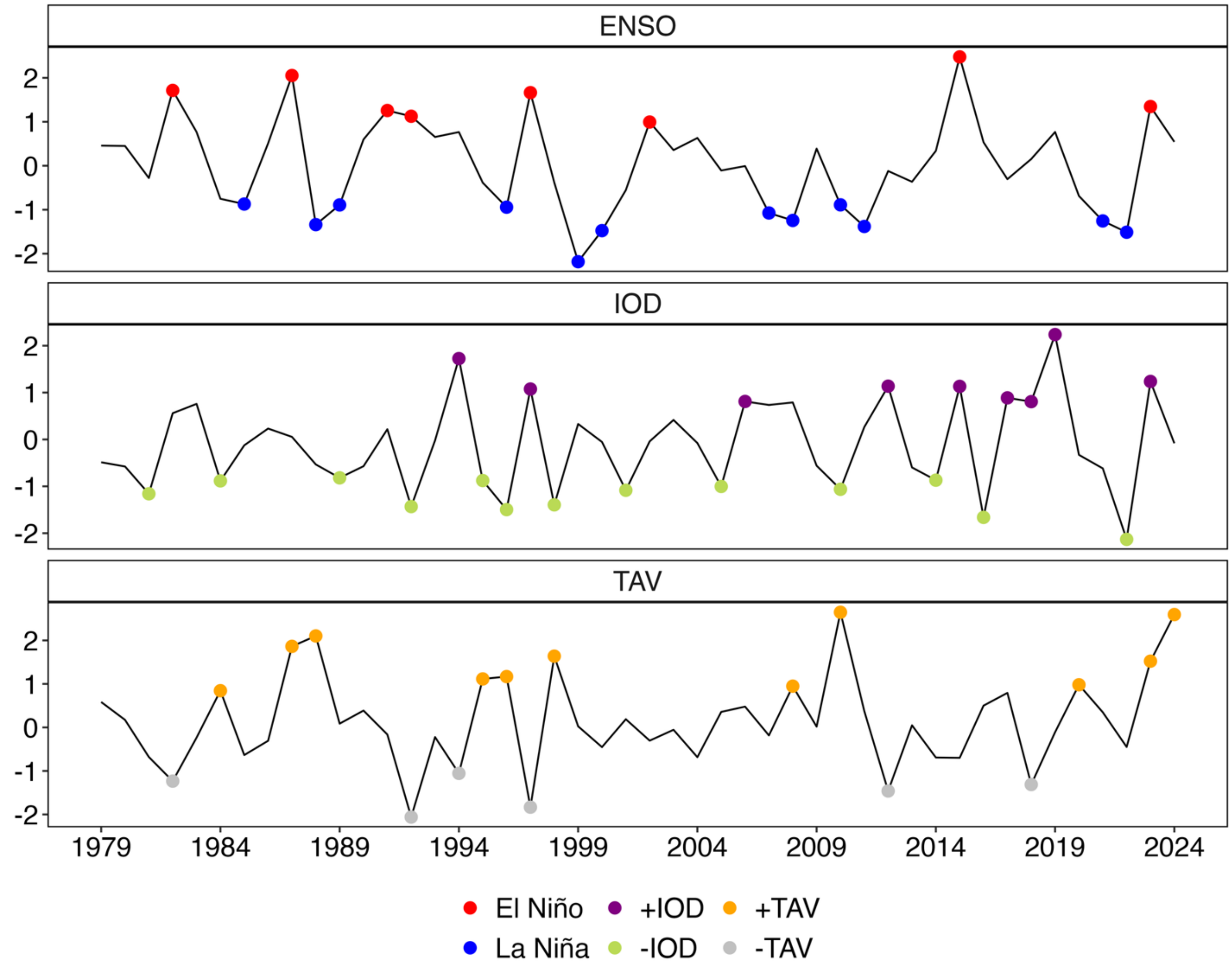

Fig. S8. Positive and negative phases of ENSO, IOD, and TAV. Time series of ENSO, IOD, and TAV indices from 1979 to 2024, along with the occurrence of El Niño, La Niña, positive and negative IOD years, and positive and negative TAV years.

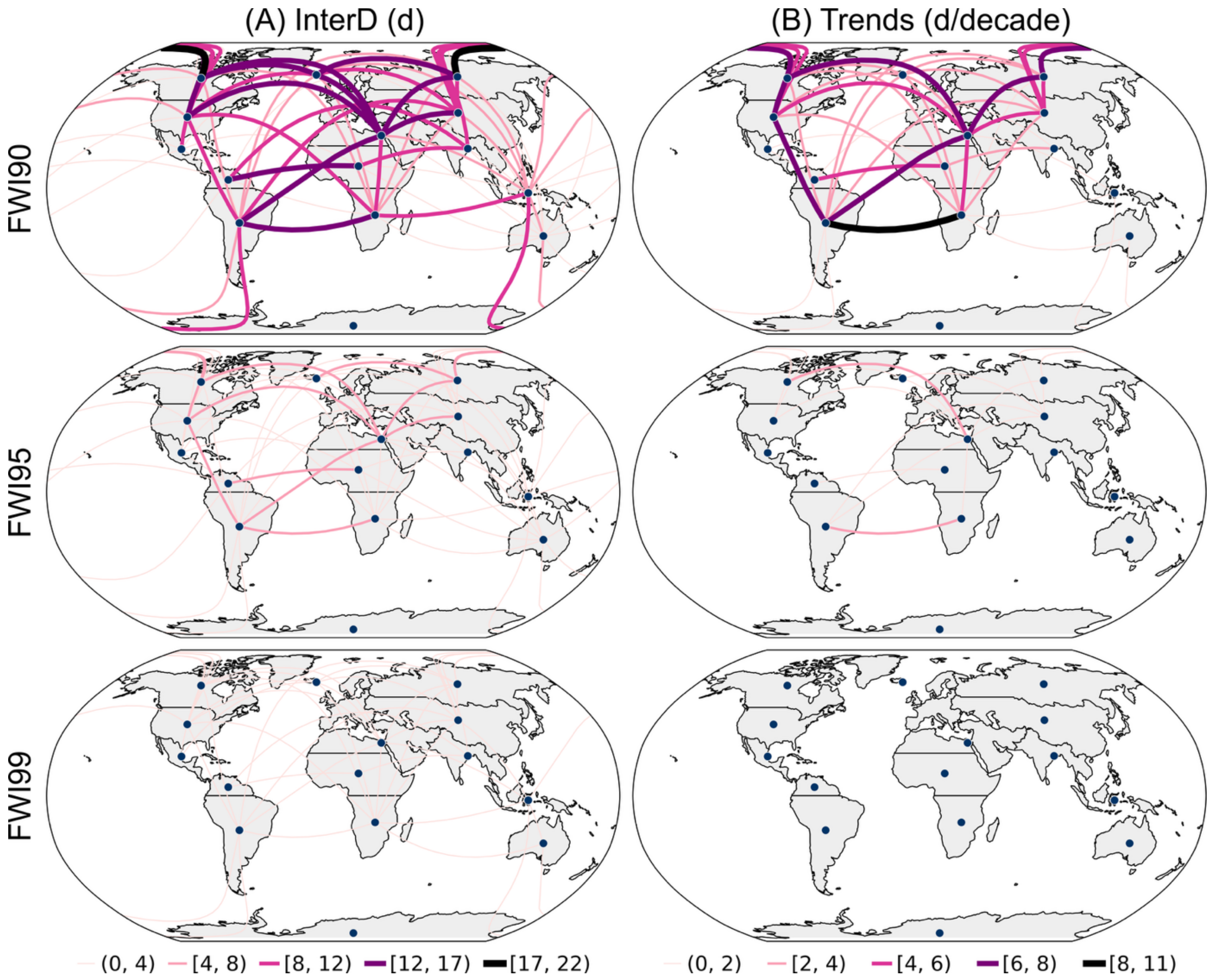

Fig. S9. Sensitivity of InterD to FWI thresholds. (A) Yearly average InterD from 1979 to 2024 between connected GFED regions, based on different FWI thresholds used to define SFW. (B) Same as (A), but showing significant trends ($p < 0.05$) in InterD.

Table S1. CMIP6 climate models used to calculate counterfactual FWI. The first ensemble member was used for each model.

Model	Institution
**ACCESS-CM2**	CSIRO & BoM, Australia
**AWI-CM-1-1-MR**	Alfred Wegener Institute, Germany
**CanESM5-CanOE**	CCCma, Canada
**CMCC-ESM2**	CMCC, Italy
**CNRM-CM6-1-HR**	CNRM-CERFACS, France
**CNRM-CM6-1**	CNRM-CERFACS, France
**CNRM-ESM2-1**	CNRM-CERFACS, France
**EC-Earth3-CC**	EC-Earth Consortium
**EC-Earth3-Veg-LR**	EC-Earth Consortium
**FIO-ESM-2-0**	FIO, China
**GFDL-ESM4**	NOAA GFDL, USA
**HadGEM3-CG31-LL**	Met Office Hadley Centre, UK
**INM-CM4-8**	INM, Russia
**INM-CM5-0**	INM, Russia
**IPSL-CM6A-LR**	IPSL, France
**MIROC6**	JAMSTEC / AORI / NIES, Japan
**MIROC-ES2L**	JAMSTEC / AORI / NIES, Japan
**MPI-ESM1-2-LR**	MPI-M, Germany
**MRI-ESM2-0**	MRI, Japan
**UKESM1-0-LL**	Met Office Hadley Centre, UK

Table S2. Sensitivity of IntraD and its trend to FWI and area thresholds.

Region	IntraD (D)					Trends (d/decade)				
	FWI90, 20%	FWI90, 30%	FWI90, 40%	FWI95, 30%	FWI99, 30%	FWI90, 20%	FWI90, 30%	FWI90, 40%	FWI95, 30%	FWI99, 30%
AUST	59.83	25.52	8.96	4.80	0.04	0.36	0.21	0.1	0.07	0
BOAS	83.67	53.02	21.96	6.87	0.00	0.75*	0.83*	0.72*	0.20*	0
BONA	87.46	49.28	17.87	4.57	0.00	0.61*	0.62*	0.25	0.09*	0
CEAM	58.11	36.78	21.07	11.43	0.35	1.12*	0.73*	0.4	0.20*	0
CEAS	56.22	12.33	1.02	0.28	0.00	1.67*	0.56*	0	0.00*	0
EQAS	47.46	24.30	12.74	8.67	0.39	-0.11	0	0	0	0
EURO	68.35	37.89	20.46	13.04	0.78	0.48*	0.23	0.07	0.02	0
MIDE	71.00	39.52	18.65	6.63	0.00	1.20*	1.36*	0.85*	0.20*	0
NHAF	63.80	40.17	22.72	12.39	0.22	1.38*	1.14*	0.68*	0.35*	0
NHSA	59.67	35.72	21.41	13.04	1.28	1.00*	0.58*	0.33	0.11	0
SEAS	66.96	35.57	16.50	8.33	0.02	-0.41*	-0.43	-0.32*	-0.21*	0
SHAF	60.02	24.74	8.24	3.11	0.00	1.74*	0.86*	0.33*	0.07*	0
SHSA	48.17	29.11	16.20	9.98	0.70	2.49*	1.74*	0.92*	0.34*	0
TENA	63.48	24.15	6.33	2.59	0.00	2.00*	0.79*	0.17*	0	0

Asterisks (*) indicate significant trends based on the modified Mann–Kendall test after FDR correction.
